# A qualitative analysis of vertical leadership development amongst NHS health-care workers in low to middle income country settings

**DOI:** 10.1108/LHS-11-2020-0089

**Published:** 2021-07-28

**Authors:** Ann-Marie Streeton, Fleur Kitsell, Nichola Gambles, Rose McCarthy

**Affiliations:** Volunteer, Global Health Partnerships Directorate, Health Education England and General Practice, Partner, Clevedon Medical Centre, Clevedon, UK; Global Health Partnerships Directorate, Health Education England, London, UK; Research Assistant, Research Assistant, Global Health Partnerships Directorate, Health Education England, Manchester, UK; Head of Faculty, Global Health Partnerships Directorate, Health Education England, Manchester, UK

**Keywords:** National health service, Global health, Global engagement, Improving global health, Leadership development programme, Vertical leadership development

## Abstract

**Purpose:**

The improving global health (IGH) programme is a leadership development programme that aims to develop leadership skills and behaviours alongside quality improvement methodology in National Health Service (NHS) employees in a global health setting. Through collaboration, experiential learning and mentorship, the programme aims to produce both vertical and horizontal leadership development in its participants. This paper aims to describe the programme and its impact, in terms of leadership development, in a sample of participants.

**Design/methodology/approach:**

Open coding and thematic analysis of leadership development summaries (LDS) completed by 39 returned IGH participants were conducted. LDS are written on completion of the overseas placement; participants reflect on their personal leadership development against the nine dimensions of the NHS Healthcare Leadership Model (2013).

**Findings:**

These IGH programme participants have reported a change in the way they think, behave and see the world. A development in sense of self and experience in developing team members are the two most commonly reported themes. Adaptability, communication, overcoming boundaries, collaborative working, “big picture” thinking and strategic thinking were also identified.

**Research limitations/implications:**

The study is limited by the relatively low number of completed LDS. More work is needed to understand the long-term effect of this type of leadership development on the NHS. Other leadership development programmes should consider focussing on vertical and horizontal leadership development.

**Originality/value:**

This more granular understanding of the leadership skills and behaviours developed and how it is the programme’s design that creates it, has not previously been described.

## Purpose and context

The National Health Service (NHS) is currently facing unprecedented challenges with, in particular, a global pandemic. In this time of rapid change, pressures and financial constraints; effective leadership is imperative to ensure patient care and safety are not compromised. The significance of leadership within the NHS has been highlighted with increasing frequency over recent years. Leadership is defined as “the ability and capacity to lead and influence others, by means of personal attributes and/or behaviours, to achieve a common goal” (NHS England and Health Education England [HEE], 2018).

High quality, compassionate and inclusive leadership must be developed at all levels of the NHS (National Improvement and Leadership Development Board, 2016), (Department of Health, 2017). To achieve this, leadership and improvement are to be embedded in curricula and revalidation throughout clinical training (Department of Health and Social Care, 2019). When NHS staff feel able to contribute to improvements at work and feel engaged; patient satisfaction, patient mortality rates and trust financial performance are better (West, 2011). Leadership positively effects hospital service quality (Elkomy, 2018). It is, however, difficult to define the behaviours of leaders as they are diverse and present in different ways (Faculty of Medical Leadership and Management [FMLM], centre for creative leadership [CCL], Kings Fund, 2015). Qualities such as self-awareness, self-confidence, self-control, self-knowledge, personal reflection, resilience and determination are thought to form the basis of our behaviour and how we interact with others. It is these interactions that form the basis of team culture, which, if more positive produces a better experience of health care for patients and their families (NHS Leadership Academy, 2013).

To optimise the quality of patient care it is vital that we support the growth of NHS leadership programmes and take the time to critically appraise them, ensuring they deliver on their promise to produce robust leaders of the future. The improving global health (IGH) Programme is one of a number of NHS leadership programmes. While the nursing clinical leadership programme has been shown to improve nurse’s transformational leadership competencies (Martin, 2012) and the London “Darzi” Fellowship reports an impact “that is far reaching” (Stoll, 2011), the leadership development created by the IGH programme has not yet been fully explored.

This evaluation explores how the thought processes and behaviours of this group of participants have changed, aiming to build a more granular picture of the nature of the leadership skills that are being developed. Evidence is presented that the IGH programme produces horizontal and vertical leadership development in its participants and that it is the programme’s design that stimulates this development.

## The improving global health programme

The IGH programme is an HEE funded programme which aims to develop leadership skills and behaviours in participants while improvements in low- and middle- income health-care systems are realised. Participants are given the opportunity to develop their leadership skills through practical application and experiential learning (Kolb, 2001) within a global health setting. They work with overseas’ health teams to coordinate six-month system-strengthening projects chosen by the in-country team. The programme is open to application from all NHS employees. The majority of participants are junior doctors, a range of other professionals are represented-managers, physiotherapists, pharmacists and nurses, to name a few. There is a written application followed by an interview with a panel made up of three IGH employees and/or IGH Alumni. During the lifetime of the programme 12 overseas partnerships have been formed with organisations in Cambodia, Myanmar, Tanzania, Kenya, South Africa, Zambia, Lesotho and Uganda. Fellows are given the opportunity to choose which partnership site best suits their needs.

Training and support are robust and multi-faceted. Pre-placement, participants complete four days of UK-based induction where they learn concepts of leadership; project planning, implementation and evaluation; quality improvement (QI) methods; peer learning and support; concepts of public health and the wider determinants of health; the UN Sustainable Development Goals and have time to complete and receive feedback regarding their Myers-Briggs type indicator. Cultural preparation is facilitated through a session delivered at induction by the partnership-link lead (PLL) for each country site and through access to IGH alumni.

Participants are allocated a mentor to support them throughout their fellowship and have access to a PLL whose main purpose is to support the relationship with the partnership site itself. Participants do not provide direct clinical care. Project topics are chosen by the in-country team, ensuring work is aligned with local health-care priorities. A project plan (submitted after one-month), a monitoring report (submitted after three-months) and an evaluation report (submitted before leaving at six-months) are all completed. Post-placement, participants complete a leadership development summary (LDS) and present their work and learning at an IGH meeting.

Previous evaluations have shown that participants develop a wide breath of leadership skills that they take back to their careers in the NHS. The ability to self-identify as a leader and the increase in self-efficacy has been shown to positively impact career direction (Monkhouse, 2018; Walmsley, 2012). International volunteering has been shown to generate average productivity gains for the NHS of up to 37% for doctors and up to 62% for nurses; for health partnerships, the gains vary depending on the duration of volunteering periods and occupational category mix (Zamora, 2019).

### Horizontal and vertical leadership development

“Leadership is becoming less about being the smartest in the room and much more about how we collaborate, work with diverse stakeholders, inspire and bring the best out of others. Being more inclusive and collaborative. It’s about developing our ability to be curious; our ability to explore new approaches, new perspectives, engage different stakeholders and view points and empathise with diverse perspectives” (Rose, 2015).

**Figure 1.**
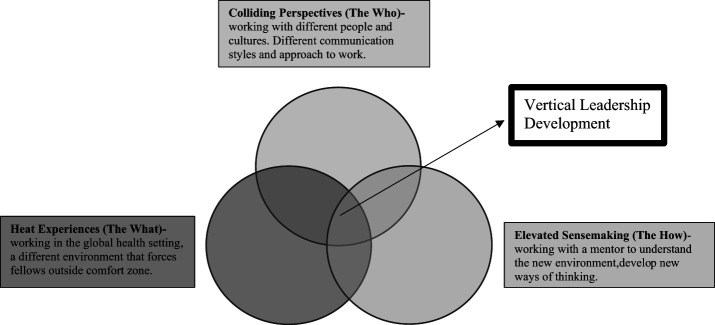
The IGH programme as a vertical leadership development programme-reproduced and adapted (Petrie, 2015)

The IGH programme grew organically. Initially, with the NHS leadership framework (NHS Leadership Academy, 2011) in mind, superseded by the NHS health-care leadership model (NHS Leadership Academy, 2013) but later with vertical leadership development in mind. Horizontal leadership development is about imparting knowledge and facts while vertical leadership development is about changing the way you think (Petrie, 2014). The IGH programme design fits well with the proposed design of a horizontal and vertical leadership development programme. As described by Nick Petrie, vertical development needs a “heat experience”, “colliding perspectives” and “elevated sense making” (Figure 1). The induction programme produces horizontal leadership development where participants gain theoretical knowledge while the six-month overseas placement where they collaborate on a QI project produces the vertical leadership development. Participants are placed in an unfamiliar environment that is very different to their own, serving as their “heat experience”; they are forced to consider new and different ways of thinking to face this challenge. Communication, experiences, culture and training are varied and contrasting, this produces the “colliding perspectives” element. Finally, they are provided with mentorship to guide, mould and coach the new thought processes, “elevated sense making” (Petrie, 2015).

The global health setting aims to intensify the “heat experience” and galvanise transformational change (Petrie, 2015). The experience of working internationally achieves outcomes of improved communication, leadership, attitude to work, flexibility and cultural awareness (Tyler, 2019). However, there is a gap in the evidence base for a link between global health work and vertical leadership development, particularly in the context of a formal leadership programme.

The modern world has been described as volatile, uncertain, complex and ambiguous, an acronym first used in 1987, drawing on the leadership theories of Warren Bennis and Burt Nanus (Bennis and Nanus, 1985). This is certainly true of health care and also the NHS. For leaders to flourish in this ever-changing system, older styles of leadership must be abandoned in favour of newer styles of leadership (Till, 2016) which promote a more positive and cohesive working environment (Belrhiti, 2018). Nick Petrie describes vertical leadership development as moving from the state of “faithful follower” where direction and belief systems are aligned with others, to “independent thinker” where the individual is self-directed and has their own internal compass, to the final stage of “interdependent collaborator” where they are able to see the whole picture with all its different perspectives and contradictions (Petrie, 2014).

### Method

Over a three-year period from February 2016 to July 2019, 90 NHS employees completed the IGH programme. Participants are asked to submit a completed LDS four-weeks after return to the UK. Participants self-assess by reflecting on their leadership development, which is mapped onto the nine domains of the NHS health-care leadership model, namely, “Inspiring shared purpose”, “leading with care”, “evaluating information”, “connecting our service”, “sharing the vision”, “engaging the team”, “holding to account”, “developing capability” and “influencing for results” (NHS Leadership Academy, 2013). They reflect on how they developed in dimensions they feel are relevant (using examples) and are asked to indicate how they might further develop. Their mentor is asked to comment on the participant’s progress and any further development suggested. Participants report on dimensions they feel are most relevant within 200–300 words per dimension.

Thematic analysis was carried out using “Saturate” an online coding resource. All the reports were coded by the first researcher; a second researcher independently coded 50% of the reports. Inductive codes were applied to the data and common themes throughout assessments were identified and collated. Illustrative quotations have been used throughout the results to highlight and evidence relevant themes (Table 1). All reports were anonymised to ensure confidentiality.

## Results

**Figure 2.**
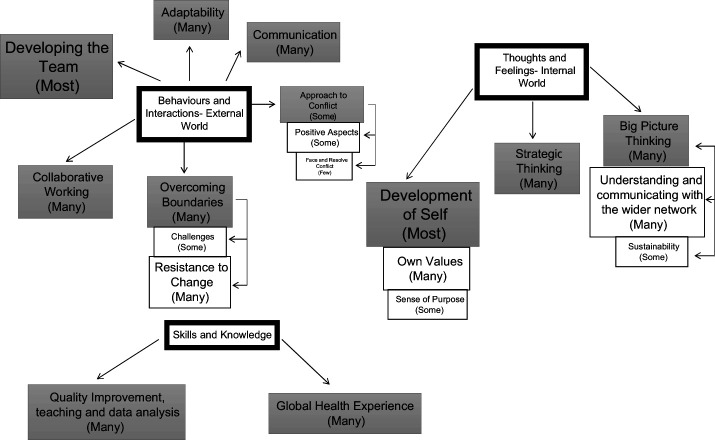
Overview of results. Orange-most, blue-many and green-some. Subthemes-clear boxes

Of the 90 participants who completed the programme, 39 submitted a completed LDS (43.3%). The results (Figure 2) have been described using the terms “a few” relating to ideas described by less than 25% of respondents, “some” relating to ideas described by 25%–50% of respondents, “many” relating to ideas described by 50%–75% of respondents and “most” relating to ideas described by over 75% of respondents.

### Behaviours and interactions-external world

#### Adaptability.

Many participants described the programme as forcing them to become more adaptable in a multitude of ways. Skills and knowledge within the local team were not predictable. Gauging the level at which to pitch teaching and knowing how to get people on board required a particularly flexible approach.

‘[…] this placement pushed me outside my comfort zone, challenged me and encouraged me to adapt to my new surroundings which [ […] ] help me enormously in the future. A great change has been my ability to act and work more flexibly. Although I always saw myself as quite a flexible worker […] really pushed this further. Working in a new environment with social, economic(al) and cultural differences put a great strain on my accustomed work style’

Participants describe using lateral thinking and imaginative solutions to overcome obstacles; thinking in this less linear way enabled them to better conceptualise solutions.

‘I re-assessed how we approached the training. I tried to creatively think of a way in which we could deliver the drills in a busy environment’.

Being forced to work outside of their comfort zone in an unfamiliar country and health system, made the need for lateral thinking more evident. Learning together across the world in low- and middle-income countries are thought to provide more scope for creativity and innovation as there is less resistance from institutions and vested interests (Crisp, 2014). An improvement in negotiation skills was mentioned, where participants felt better able to accept a difference in opinion and find compromise.

‘[…] better at accepting and welcoming varying opinions and looking for common ground rather than just attempting to change their opinion to more similar to mine’.

### Approach to conflict

Some participants described feeling better at anticipating and facing conflict, both with other UK fellows with whom they shared living space, social life and work; and with the in-country team. Overall, they learnt that conflict must sometimes be faced and embraced to bring authenticity to their working relationships.

#### Positive aspects.

Some participants discussed the positive aspects of conflict, recognising how mood and communication can affect relationships. Anticipating conflict and responding early allowed the development of supportive relationships which, in turn, produced a healthier working environment.

‘[…] our working relationship was hardly ever strained and we both worked to anticipate situations that could be problematic and prophylactically manage it. This approach to encourage a health(y), positive and supporting working environment is one that I have now taken forward […] my new role in the NHS’.

Participants identified conflict as an opportunity for change. Where they previously judged themselves on feedback from others, improved self-awareness meant conflict could be seen more positively through choosing to focus on qualities while accepting there is no right approach.

‘[…] I recognised that my tendency to closely link my ideas to my perception of myself, and whether I am ‘successful’ was […] negative in enabling me to take criticism on board. Gaining deeper self-awareness of issues such as these helped me to respond more positively […] it was important to try to remember that it does not matter who is ‘right’- the important thing is finding some consensus from which to move forward […]’

##### Face and resolve conflict.

A few participants described feeling better at resolving conflict. Understanding the cause of disagreement meant that it was easier to empathise; sometimes it is best to let things go and avoid conflict to preserve relationships and maintain a positive working environment. Reflecting and learning meant becoming better able to cope with similar future scenarios.

‘Choosing when not to create conflict, after appraising the culture of the organisation, was as important as choosing which causes were worth applying pressure for. Preserving a relationship, not causing someone to lose face and maintaining positive teamwork would ensure that longer term success of the strategy […] compared to creating a climate of animosity’.

### Communication

Many participants talked about an improvement in their ability to communicate. There were language barriers, triggering the development of non-verbal approaches– diagrams or written communication. Participants planned to teach around the timetables of their colleagues and scheduled regular sessions to enable repetition of concepts. Working in a new environment offered a safe space to work on softer skills. Participants report improvement in focussed listening, understanding and ability to adapt to meet the needs of a varied audience. Working with different cultures made them consider how culture affects communication and also leadership. Leadership style is thought to be culturally linked (Jogulu, 2010).

‘In an NHS setting I have developed a sense of the tools that can be used to influence people, and how far I can push people before coming across as rude or insensitive […] I didn’t […] ha(ve) the same sense in […] This was something I was able to develop […] during the placement […]’.

IGH programme induction teaches participants different approaches to influencing, (Musselwhite and Plouffe, 2012) while the in-country placement gives fellows time to practice. Effective leaders understand the way others want to be influenced and apply the right tactics to build alignment and commitment (Dellaert and Davydov, 2017).

‘I also benefited from the individualised, objective feedback on my influencing styles […] very helpful and I have set the objective to develop my ‘pull’ influencing style back at work’

Participants also learnt how to deliver and receive feedback. They found it hard to deliver negative feedback, but with time became more comfortable with being honest. The difficulty was expressed in navigating cultural norms, some cultures were not used to feedback. Participants were motivated to create locally appropriate solutions e.g. delivering anonymised group feedback.

‘For the staff members I see on a weekly basis I take the time to talk to them one-on-one and give them positive feedback and constructive areas where they could improve’

Other comments were that communication could be unreliable between local organisations, resulting in the overlap between the work conducted by different sectors and that it could be difficult getting the local team on board as an outsider.

Communication between sectors and disciplines […] was not as effective as it could have been […] I was able to witness the critical nature of this aspect in achieving the best results most efficiently.

International volunteering in low-income settings improves communication skills (Tyler, 2018).

### Overcoming boundaries

Many participants learnt to overcome boundaries, the most frequently quoted being communication and culture. Participants chose to spend time shadowing the local team during work shifts and building relationships. They asked questions and listened. Fellows developed “deep listening skills” (Petrie, 2015), they took the time to focus on the content, emotions and values of their colleagues.

‘It has been challenging, particularly as a foreigner, coming into a country that is currently undergoing huge transition […] working with an organisation which is not yet fully established and on a project that aims to integrate them into a system which they have been excluded […] for years, has been an ambitious undertaking’.

#### Challenges.

Some participants explained that they learnt to challenge hierarchy and the “status quo”. Questioning authority and learning to appropriately escalate problems initially felt uncomfortable but with time and practice became easier. Speaking up in the NHS is something that everyone is encouraged to do (Francis, 2014).

‘The culture locally dictates that one does not challenge or question someone senior. As a foreigner, one doesn’t fit into their local hierarchy […] I was able to ask questions and suggest an alternative to a certain degree […] the balance between having a discussion and being seen as a direct challenge was difficult’

Participants describe learning from challenges and becoming better at accepting set-backs. “Real world challenges”, part of the experiential learning of the IGH programme, are known to be more vivid in the global health context (All-Party Parliamentary Group on Global Health, 2013). Participants discuss the experience and learning gained from working with different cultures, examples of the development of cultural intelligence. Cultural and emotional intelligence has been shown to be positively correlated. Cultural intelligence is a competency that improves leader and team performance, particularly within ethnically diverse work environments (Groves and Feyerherm, 2011).

‘[…] being placed in such a differing environment challenged even my more competent leadership skills and gave me opportunities to be tested and to develop. I can’t picture an environment in the UK or NHS that could challenge me this much and provide such wide-ranging experiences’.

Feeling judged due to seniority, experience, age and/or gender was also reported. This experience of bias provides an opportunity to better understand how discrimination might feel, pushing fellows towards a more inclusive style of leadership which is known to improve productivity, satisfaction and engagement (Bucks New University, 2016).

‘[…] she was underestimated initially due to being young […] the relevance and power of these factors should not be disregarded even in the NHS’.

#### Resistance to change.

Many participants encountered resistance to change. A positive attitude reportedly helped, as did provide training/support so that the team did not feel overwhelmed or intimidated.

‘There appears to be a default stance of resistance to change and is multifactorial based on which staff group it comes from […] the nursing staff […] were not overtly resistant, but scared of what they saw as such an advanced system they were not fully IT literate’.

There was inherent difficulty in running a project in an environment where the participants were not known. This difficulty could be used to their advantage, as they were more able to ask the “difficult questions”.

‘Trying to engage the wider […] team was a crucial aim for me […] by working collaboratively, we could all achieve much more- but this dynamic was challenging because we did not lead the health team. In the end we used our role as ‘outsiders’ to bring people together’.

### Collaborative working

Many participants learnt to work collaboratively, describing the importance of listening, recognising the contribution of individuals and celebrating achievements; and how this enables the building of positive relationships.

‘[…] she recognises those who have worked hard and provides encouragement and celebrated achievement of her team as part of her leadership role […] helping to build even more constructive and open relationships with team members’

Living, working and socialising with other IGH fellows, often having never met prior to the start of their project, was described as complicated.

‘[…] as a group of IGH fellows we worked well together as from the beginning were open about our strengths and weaknesses and would often discuss our work together to share ideas’

The experience was gained in bringing different organisations and departments together. Sharing with the wider team enabled work to be more locally relevant and embedded. In this way the participants are developing a more collective or shared leadership style where they are working across organisational boundaries, building relationships with team members and recognising the power of the individual team member. Leaders of the NHS are encouraged to focus on a more collective leadership style (FMLM, CCL, Kings Fund, 2015). Shared leadership positively relates to team goal attainment (Bienefeld and Grote, 2014).

‘[…] we focused on […] increasing the connection between different […] departments and I feel we had a very positive result here. (The organisation) has a wealth of information in its different departments and the areas they work in are very interlinked; however, they were working in relative isolation. We arranged a number of cross-departmental meetings […] to encourage […] departments to link further and discuss […] ’

Participants often mentioned managing different perspectives and agendas. Participants explained that their local colleagues had been trained differently, had different experiences of clinical medicine and that local bureaucracy worked differently. Therefore, time had to be spent understanding before progress could be made.

‘[…] consider and understand what motivates different individuals to work out the best ways to engage them. Before my placement, I was very aware of how (team leader) might feel about two […] coming to work in his team and developing an overall strategy for what is effectively his work. I also thought about how from his point of view, having new UK fellows every 6-months could be quite tiring’

Participants spent time creating a no-blame culture where team members were able to voice opinions without fear of reproach, in keeping with today’s leaders of the NHS who are expected to be more “humane and approachable” than in the past (Anandaciva, 2018). Participants learnt from the local team by working closely, sometimes observing inspiring leadership. It is this cycle of experience, reflection, conceptualisation and experimentation that produces the experiential learning that is the crux of the IGH programme (Kolb, 2001).

‘It was an honour and a privilege to be welcomed into a community and to be given so much of people’s time and experience their goodwill. The number of inspirational leaders I encountered was astounding […] I feel it must be something about the personality of the […] people, their experiences, triumphs and struggles that has led so many people to be so passionate and determined for change’

#### Developing the team.

Most participants described developing the local team in some way. The local team developed their careers through a direct enhancement in their skills from teaching and mentorship.

‘I encouraged her to think about the longer term, rather than just the immediate future- to consider what she wants to be doing in say 5 years and how best she could structure her career to get her to that point’

The participants recognised the need for accountability and autonomy in their team members, a key aspect of staff engagement. When staff engagement scores are low, standardised mortality rates for patients are higher (Storey and Holti, 2013). Building autonomy within team members promotes a collective leadership approach, which has a positive effect on team effectiveness (Wang, 2014).

‘[…] there were several times where I felt I needed to remind people that they were yet to deliver what had been agreed. It was a very different environment to develop this skill because the challenges were different.’

Other areas mentioned were teaching QI methodology and inspiring the local team.

‘[…] communicating information honestly and boosting morale by ensuring all members of the team were aware that they were appreciated and that their work was important, I wanted to take this further […] by making people really believe that change (in whatever guise) was possible’

Inspiring colleagues to learn and develop gave them the impetus to feel that change was possible and that systems can and would improve; an example of transformational leadership where as follows: “leaders and followers make each other advance to a higher level of morality and motivation” (Burns, 1978).

‘[…] are inspirational leaders with a joie de vivre and absolute conviction in their ability to change things for the better, in a health system even more resource constrained and troubled then our own’

## Thoughts and feelings-internal world

### “Big picture” thinking

#### Understanding and communicating with the wider network.

Many participants describe a better understanding of the whole health-care system, seeing the system as complex and changeable rather than closed and controllable. Participants gained experience and knowledge of the overall structure of health-care systems not only at the level of public health, primary care and secondary care but also at the level of government and politics.

‘[…] getting a better understanding of how the health care system is organised […] I got to visit many hospitals and speak to […] healthcare professions but even at the end I am sure I did not have a full understanding of the different system infrastructures’

The opportunity to attend meetings with hospital management, organise their own meetings, understand budgets and learn about governmental level politics were also mentioned. As leaders of the future participants will need to understand corporate politics and know how organisations function (Dellaert and Davydov, 2017). Participants felt they would have struggled to gain this experience within their NHS roles and that this experience is unique to the global health aspect of this program.

‘[…] I can’t picture an environment in the UK or NHS that could challenge me this much and provide such wide-ranging experiences’.

#### Sustainability.

Some of the participants reported learning about sustainability within QI work generally and more specifically in global health. They describe affecting longer-term change by developing skills, knowledge and leadership within the local team.

‘A big part […] was to create something that would be sustainable, so that it would continue to benefit the host country even after we have left […] I realised the importance of setting goals for the individual GPs I was working with, challenging them, but with a supportive environment so that their new skills would continue to be of benefit in the future’.

One participant commented on the use of the health partnership model (Crisp, 2008) enabling the fostering of long-term relationships between IGH and the local team ensuring mutual respect and sustainability.

‘I was attracted to the IGH programme because of the emphasis on the sustainability of the work achieved by long-term partnerships and working on projects that are developed and directed by the local team’.

### Strategic thinking

Many participants gained experience in thinking strategically. They brought local teams together to work towards a unified goal by setting expectations and communicating a clear vision despite opposing needs.

‘[…] found it quite difficult initially to feel confident in […] identifying what the main goals were as I didn’t feel I truly understand my environment and the problems it faced therefore I could not make a clear vision. After a few months, I began to develop a key theme and vision for all my work […] ’

Many participants describe the importance of stakeholder engagement, needing to appease multiple stakeholders and cope with competing interests within the same system.

‘[…] I worked to try and improve this (engagement with the new health strategy) throughout the placement- involving him with strategy developing and trying to ascertain his thoughts and ideas. Whilst he engaged to a certain degree with some aspects, at the time we left […] was yet to read the (report)’

### Development of self

Most participants felt they had developed their sense of self in some way

#### Own values.

Most commonly participants described becoming more confident and/or assertive. They felt more able to approach people in positions of power, accept and hold a difference in opinion and voice concerns. Participants felt the improvement in their confidence was retained on return to the UK and in some instances helped them apply for more senior roles.

‘It has given me the confidence to apply for an MBA apprenticeship within my trust which I have just started. My journey continues!’

Participants also felt the programme gave them time to think about who they are as a person, in particular, what their own limits, goals and values are.

‘It requires a great deal of self-insight […] has made me more aware of my capacity and my limitations

Participants described feeling more emotionally intelligent. They felt more “in tune” with their emotions, and had a better understanding of how their emotions can affect other people. They gained a deeper sense of self, feeling more able to let things go and look for consensus opinion rather than worrying about “who is right”. As outlined by Goleman, the five essential elements of emotional intelligence are emotional self-awareness, self-regulation, motivation, empathy and social skills. Having emotional intelligence is essential to the success of leaders (Goleman, 2011). The need for cognitive and interpersonal development within leaders of complex systems such as the NHS has been outlined (Petrie and Swanson, 2018).

#### Sense of purpose.

Some participants described improved perseverance, resilience and enthusiasm, examples of “psychological capital”. Psychological capital can be drawn upon when needed by leaders to respond effectively to the pressure, demands and responsibility of authority (Ruderman and Clerkin, 2015). participants were able to consider what they wanted from life and work; and had time for reflective thinking.

‘Listening to people and all stakeholders, asking open questions which are not leading, not interrupting and saving questions till the end. Patience and persistence […] that coming to a consensus takes time and requires time for people to think’.

As vertical leaders develop, sense making becomes more sophisticated and nuanced resulting in the development of wisdom. Wisdom leadership is necessary for leading change in complex organisations (Pesut and Thompson, 2018).

## Skills and knowledge

### Global health experience

Many participants felt they had a better understanding of the resource poor context of the low- or middle-income country in which they were working. Participants went on to explain that they would like to take this global experience and the learning they have gained back to their work in the NHS.

‘The environment that […] colleagues work in is difficult to understand without experiencing it and being exposed to it […] I(t) was […] interesting to gain an understanding over a 6-month period, and I enjoyed seeing how my thoughts and opinions changed over that time’.

One participant explained the negative aspects of this immersive experience.

‘Being challenged to work in a different context did sometimes have its downsides, as sometimes I felt too far out of my depth that I could not formulate events into an effective learning experience’.

Four stages are needed to create a sense of belonging in participants who complete international placements-orientation, adjustment, contribution and belonging (Wijbenga *et al.*, 2020). The IGH programme compares favourably due to its provision of an induction programme, in-country orientation and local mentorship and; the planning and implementation of a local project; although more could be done.

### Quality improvement, teaching and data analysis

Many participants reported learning new technical skills, how to collect and analyse data, run an audit project, conduct research and write academic papers.

‘[…] a really good opportunity to explore the role of data. Many of the projects that we as a team were involved in involved data collection and one of the teaching sessions that we developed as part of the QI training programme focussed on data.’

Participants gained experience in delivering teaching both formally and informally.

## Discussion

A detailed picture of the type of leadership skills and behaviours developed by these fellows has been presented. The most common areas of growth were a perceived development in “sense of self” and “team members”. These participants describe learning to face challenges, deal with ambiguity and “share knowledge across boundaries”; they are learning to “see the world through others perspectives to understand more”, show a more sophisticated understanding of “how the different layers of health systems come together” and are starting to see success in leadership as “the realisation of a shared vision” (Petrie, 2014). Overall, evidence has been presented that the leadership development of this group of fellows is consistent with the final stage of vertical leadership development, “the interdependent collaborator” (Petrie, 2014). Horizontal leadership development has also been achieved through an improvement in QI methodology and global health knowledge.

Leaders of this nature who can think independently, adapt and be open to change are ever more needed in the current climate of a rapidly changing NHS, strained by the demands of a global pandemic. Leaders who are more culturally and emotionally aware will be more at home working and communicating globally. In so doing, communities can be brought together both not only within global health but also within a culturally diverse NHS. It is vital that knowledge and practice is shared globally, the pandemic is an exemplar of this. As the global health community grows, the potential for learning and information exchange grows with it. The skills and behaviours developed by fellows on this programme have a long-lasting effect on career direction (Streeton *et al.*, 2021), suggesting the leadership potential created is also long-lasting. A new calibre of NHS leader is being created, independent of thought, open to change and understanding of difference, all attributes that will help the NHS respond to its current pressures. As a response to the global pandemic, the IGH programme is piloting remote virtual fellowships to which fellows have already been recruited. This pilot builds on learning from a mixed model of pharmacy fellowships on anti-microbial stewardship supported by the Fleming fund, the Commonwealth Association of Pharmacists, the tropical health and education trust and HEE and is an example of the type of adaptation that is needed to enable programmes such as the IGH programme to continue to grow and thrive in an extremely challenging time.

The use of LDS in this evaluation has both strengths and weaknesses. Self-reflective data was captured as soon as the fellowship had been completed, rather than months or years later when participants may not have remembered detail or may have had other experiences that interfere with self-reported development from the programme. The study is, however, limited as less than half of returned IGH participants completed and submitted their leadership reports; likely due to time pressure on the returned participants from busy jobs and re-engaging with life in the UK. As the summaries are prescriptive, it was not possible to question and delve deeper as would have been possible with an interview approach; the level of detail provided in these reports is variable, impacting on the overall quality of programme evaluation.

## Recommendations

Further work is needed to improve understanding of the depth of vertical leadership development created by this programme and how this relates to its global health approach. An evaluation of a larger number of returned participants using an interview approach is recommended. Work is needed to ascertain if this leadership growth is sustainable and reproducible by other programmes, also if this type of leadership development ultimately leads to improved NHS patient outcomes. Better training of participants on how to complete these reports at induction along with more support from mentors and PLL may help. A system of ongoing programme evaluation and feedback should continue to inform development decisions, particularly as the programme grows, making management and reporting more challenging.

## Conclusion

Leadership within the NHS is a burgeoning field. There are many leadership development programmes currently available to NHS employees, all with different structures and approaches. The IGH programme is unique in its design as it provides the opportunity for a multi-disciplinary group of NHS workers to learn leadership and QI theory, and then apply this theory to collaboratively leading projects within a global health setting. It is the structure of the programme, not only the use of the overseas placement but also the use of an intensive induction programme, the availability of support through mentorship and also the encouragement to reflect that stimulates leadership growth in the participant.

We are going through an era of rapid change, where managing a global pandemic and adapting to work with larger and more diverse teams while being mindful of ongoing financial constraints has become the norm. The health-care clinicians created by the IGH programme who are more mature, adaptable and culturally aware are now, more than ever, relevant. This evaluation provides evidence of both vertical and horizontal leadership development in this group of fellows, although more work is needed to understand the longer-term effects of this learning and how this translates to NHS outcomes.

## Figures and Tables

**Table 1.** Overview of overarching themes, themes and subthemes

Overarching themes	Themes		Sub-themes	
1. Behaviours and interactions-external world	1.1	Adaptability		
	1.2	Approach to conflict	1.2.1	Positive aspects
			1.2.2	Face and resolve conflict
	1.3	Communication		
	1.4	Overcoming boundaries	1.4.1	Challenges
			1.4.2	Resistance to change
	1.5	Collaborative working		
	1.6	Developing the team		
2. Thoughts and feelings-internal world	2.1	“Big picture thinking”	2.1.1	Understanding and communicating with the wider network
			2.1.2	Sustainability
	2.2	Strategic thinking		
	2.3	Development of self	2.3.1	Own values
			2.3.2	Sense of purpose
3. Skills and knowledge	3.1	Global health experience		
	3.2	Quality improvement, teaching and data analysis		
